# Application Strategies of Bone Marrow Mesenchymal Stromal Cells in Bone‐Related Diseases

**DOI:** 10.1111/cpr.70178

**Published:** 2026-02-03

**Authors:** Xuemei Long, Dan Tan, Qianke Tao, Qiaonan Ye, Luwen Ye, Qing Li, Jingang Xiao

**Affiliations:** ^1^ Department of Oral Implantology, The Affiliated Stomatological Hospital Southwest Medical University Luzhou China; ^2^ Luzhou Key Laboratory of Oral & Maxillofacial Reconstruction and Regeneration, The Affiliated Stomatological Hospital Southwest Medical University Luzhou China; ^3^ Department of Periodontal and Mucosal Diseases, The Affiliated Stomatological Hospital Southwest Medical University Luzhou China; ^4^ Department of Plastic and Burn Surgery, The Affiliated Hospital Southwest Medical University Luzhou China; ^5^ Department of Oral and Maxillofacial Surgery, The Affiliated Stomatological Hospital Southwest Medical University Luzhou China

**Keywords:** bone marrow mesenchymal stromal cells (BMSCs), bone‐related diseases, osteogenic differentiation, therapeutic strategies

## Abstract

Bone‐related diseases (e.g., osteoporosis, osteoarthritis and fractures) exhibit a rising global incidence, imposing significant burdens on both quality of life and healthcare systems. Conventional therapeutic approaches, including anti‐resorptive drugs and surgical interventions, face limitations such as long‐term medication requirements, adverse effects (e.g., bisphosphonate‐related osteonecrosis of the jaw) and suboptimal efficacy. Bone marrow mesenchymal stromal cells (BMSCs) have emerged as a promising therapeutic alternative due to their accessibility, multi‐lineage differentiation potential, immunomodulatory properties and homing capacity. However, challenges such as disease complexity, mechanistic heterogeneity and therapeutic inconsistency hinder their clinical translation. Recent advances in genetic engineering, preconditioning strategies, bone tissue engineering (e.g., three‐dimensional [3D] scaffolding), extracellular vesicle‐based therapies and epigenetic regulation (e.g., histone modification) have significantly enhanced the therapeutic effects of BMSCs. Furthermore, cutting‐edge technologies like organoids and 3D bioprinting, which stem from advances in tissue engineering, offer novel avenues for clinical applications. Given these rapid developments, this review systematically summarises BMSC‐based treatment strategies for bone‐related diseases, discusses current challenges and outlines future directions to advance translational research.

## Graphical abstract

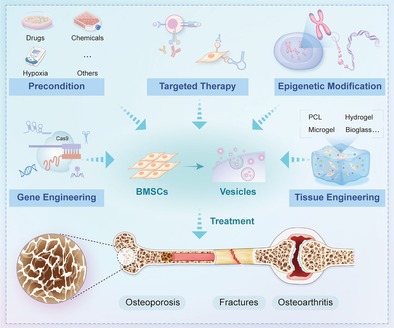

## 1 Introduction

Bone‐related disorders—including osteoporosis, osteoarthritis (OA), fractures and bone defects—are increasingly prevalent worldwide and are characterised by chronic pain, physical disability and functional impairment, imposing profound socioeconomic impacts [1, 2, 3]. Current treatments remain unsatisfactory. Osteoporosis is prevalent among the elderly, postmenopausal populations and long‐term medication users. Current treatment methods primarily managed with pharmacological interventions, including anti‐resorptive drugs (e.g., bisphosphonates and denosumab) and anabolic agents (e.g., teriparatide). Nevertheless, these regimens are hampered by prolonged treatment durations, adverse effects (including drug‐induced osteonecrosis) and suboptimal efficacy [4]. Fractures, often caused by trauma or related to osteoporosis, frequently necessitate surgical fixation but are complicated by nonunion, delayed healing or infections [5]. OA, a degenerative joint disease, lacks disease‐modifying therapies, with end‐stage cases requiring joint replacement [6]. Given the limitations of existing therapies, research has increasingly focused on elucidating the underlying mechanisms of bone diseases. This has spurring the development of advanced therapeutic strategies, notably cell‐based therapies and tissue engineering approaches, which hold promise for achieving more effective regeneration and repair.

Bone marrow mesenchymal stromal cells (BMSCs), first described by Friedenstein [7], are widely used in clinical research on bone and joint diseases. BMSCs can be efficiently isolated through bone marrow aspiration or marrow separation techniques, with well‐established in vitro culture protocols that demonstrate operational simplicity and robust expansion capacity [8]. As multipotent stromal cells, BMSCs possess three defining characteristics crucial for bone repair. First, BMSCs can differentiate into osteoblasts and chondrocytes under specific inductive cues, which makes them a pivotal cell source for bone repair [9, 10, 11]. Second, BMSCs have immunomodulatory functions, alleviating the inflammatory microenvironment in bone diseases by secreting anti‐inflammatory factors and regulating immune cell activity [12]. Third, BMSCs demonstrate homing and migration capabilities, enabling them to target and be recruited to bone injury sites through chemokine receptor‐mediated directed migration, thereby promoting tissue repair [13, 14]. These attributes underpin the central role of BMSCs in maintaining bone homeostasis, counteracting pathological bone loss and promoting regeneration [15, 16, 17].

To overcome therapeutic efficacy of BMSCs and address challenges such as inconsistent outcomes in vivo, multiple intervention strategies have been explored, including Gene Editing Technologies (e.g., CRISPR‐Cas9), Cell Engineering Approaches (e.g., pharmacological/biochemical preconditioning), Tissue Engineering Solutions (e.g., advanced biomaterial scaffolds), Derivative Therapeutics (e.g., extracellular vesicle‐based therapies), which have significantly expanded therapeutic potential of BMSCs [18, 19, 20, 21, 22]. Furthermore, emerging technologies including organoids, single‐cell sequencing and organelle research are revolutionising the field of regenerative medicine [23, 24, 25]. The cross‐integration of these technologies not only enhances the functionality and targeting capabilities of BMSCs but also broadens their application scope in treating conditions such as osteoporosis, OA and bone defects.

In this review, we summarise the current landscape of BMSC‐based therapeutic therapies, focusing on their specific applications in bone‐related diseases, explain the known mechanisms and principles and discuss the current application bottlenecks hindering clinical translation, as well as outline the future prospects of BMSC‐based regenerative medicine.

## 2 Research Foundation of BMSCs

**FIGURE 1:**
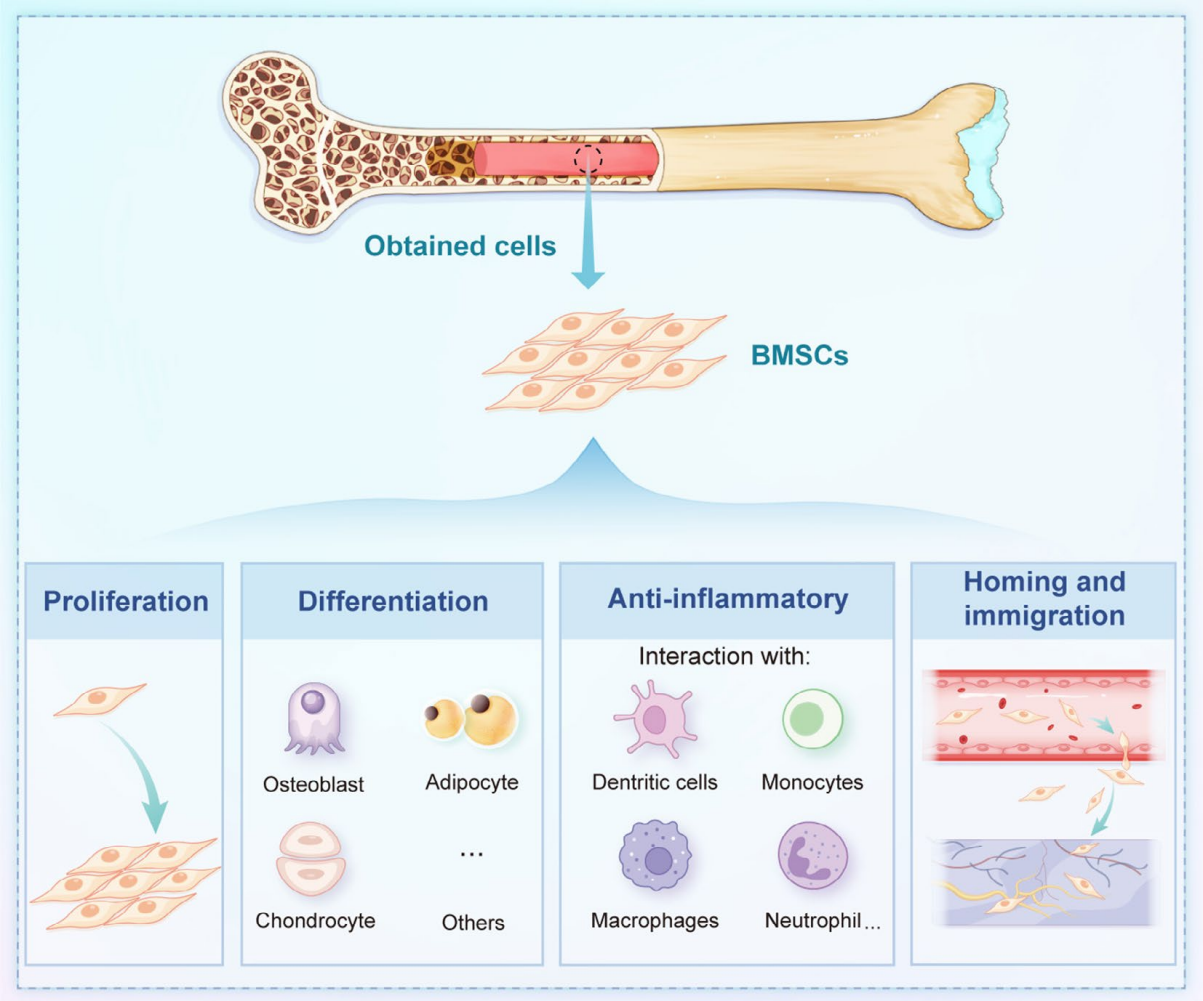
Bone marrow mesenchymal stromal cells possess the capabilities of proliferation, differentiation, anti‐inflammation, migration and homing.

BMSCs represent a cornerstone of regenerative medicine for skeletal disorders. Their multifaceted functional properties—including self‐renewal capacity, multilineage differentiation potential, immunomodulatory activity and targeted homing ability—collectively form the biological basis for their therapeutic applications (Figure 1).

### 2.1 Isolation and Characterisation of BMSCs

BMSCs are primarily isolated using bone marrow adherence, density gradient centrifugation, or bone digestion. Among these, the bone marrow adherence method is the most prevalent due to the inherent plastic‐adherence property of BMSCs and its relative operational simplicity. Briefly, bone marrow aspirate or flush are cultured under sterile conditions in Dulbecco's Modified Eagle Medium supplemented with 15% foetal bovine serum, 100 U/mL penicillin and 100 U/mL streptomycin. Cultures are maintained at 37°C in a humidified atmosphere containing 5% CO_2_. Non‐adherent cells, primarily of haematopoietic lineage, are removed by sequential medium changes. After approximately 2 weeks, a homogeneous population of adherent, spindle‐shaped BMSCs are obtained. Subsequent passaging via trypsinisation enables the expansion of BMSCs to large‐scale quantities for experimental use [26]. Given the coexistence of haematopoietic cells in bone marrow aspirates, phenotypic characterisation is essential. The identity of the isolated cells was defined according to the latest International Society for Cell & Gene Therapy (ISCT) consensus criteria for mesenchymal stromal cells (MSCs). The cells consistently expressed the core positive markers (CD73, CD90 and CD105, with > 95% positivity) as well as additional markers including CD10, CD29, CD44, CD166 and HLA‐I. They were strictly negative for the essential haematopoietic lineage marker CD45 (with < 2% positivity) and also tested negative for other lineage markers such as CD14, CD34, CD19 and CD31, confirming the absence of haematopoietic and endothelial cell contamination [27].

Conventional surface‐marker‐based characterisation, however, fails to fully capture the heterogeneity of BMSCs, which arises from genomic, transcriptomic and epigenomic diversity among individual cells [28]. Recent advances in single‐cell RNA sequencing have enhanced the characterisation of BMSCs by enabling analysis of gene expression at single‐cell resolution, genetic comparisons between individual cells and identification of distinct cellular subpopulations [29, 30, 31]. Furthermore, scRNA‐seq allows for tracking dynamic gene expression changes during in vitro culture, differentiation, or post‐transplantation, revealing the origins of heterogeneity and differentiation pathways to optimise culture and induction protocols [32, 33].

### 2.2 Proliferation and Differentiation Capacity of BMSCs

The ability of BMSCs to expand stably in vitro makes them an ideal platform for both fundamental research and clinical translation. However, their proliferative activity is influenced by multiple factors, such as physical and chemical factors in vitro culture conditions: including culture medium composition (e.g., serum type and growth factors), oxygen partial pressure (e.g., normal oxygen and low oxygen), substrate stiffness (e.g., matrix elasticity mimicking bone marrow niche) and operational influences (e.g., freezing and thawing protocols) [34]. Furthermore, replicative senescence during prolonged culture leads to reduced proliferation rates and altered differentiation potential [35, 36]. These factors pose significant challenges for large‐scale expansion, particularly in standardising quality control and maintaining genomic stability during large‐scale expansion, which directly determines therapeutic efficacy.

The osteogenic differentiation of BMSCs represents a fundamental process in bone regeneration and homeostasis maintenance, involving coordinated molecular mechanisms that include pathway activation, osteogenic gene and protein expression and epigenetic regulation. Key signalling pathways including Wnt/β‐catenin, TGF‐β/Smad, PI3K/AKT and MAPK are activated by osteogenic stimuli, promoting the expression of osteogenic‐related genes [37, 38, 39, 40, 41, 42, 43]. This cascade upregulates the production of osteogenic markers, including runt‐related transcription factor 2 (*Runx2*), Osterix, alkaline phosphatase (*Alp*), type I collagen (*Col1a1*), osteopontin (*Opn*) and osteocalcin (*Ocn*), which collectively facilitate matrix maturation and mineralisation [44, 45]. Beyond classical pathways, recent research highlights the critical roles of epigenetic modifications (e.g., DNA methylation, m^6^A RNA methylation and histone modifications) and non‐coding RNAs (e.g., miRNAs and lncRNAs) [46, 47]. For instance, studies have shown that m^6^A methyltransferase METTL3 enhances osteogenesis by modifying key transcripts and activating the Wnt/β‐catenin pathway, offering a promising therapeutic target for conditions for osteoporosis treatment [38].

BMSCs can undergo adipogenic differentiation under specific induction conditions, characterised by lipid droplet accumulation and expression of adipogenic markers like *Pparγ* and *Cebpα* [48, 49, 50]. This process holds substantial research and clinical relevance, as the resulting adipocytes secrete factors including adiponectin and leptin, enabling studies of lipid metabolism, inflammatory responses and the regulation of insulin sensitivity and glucose homeostasis [51, 52]. Furthermore, adipogenically differentiated BMSCs support soft tissue regeneration and have been applied in the repair of adipose tissue defects and breast reconstruction [53, 54]. Notably, a critical inverse relationship exists between osteogenic and adipogenic differentiation. In conditions of obesity, aging, or osteoporosis, the osteogenic differentiation capacity of BMSCs decreases while adipogenic differentiation increases. This imbalance is considered a key pathological mechanism in bone loss [55, 56]. Thus, regulating the balance between these lineages—for instance, by inhibiting *Pparγ* or activating *Runx2*—offers a promising strategy to restore bone homeostasis and inform new therapies for osteoporosis [57, 58, 59].

Under chondrogenic induction within a 3D culture environment, BMSCs differentiate into chondrocytes—cells synthesise type II collagen and proteoglycans, which are essential for cartilage matrix formation and the repair of damaged cartilage [60]. This chondrogenic potential provides a promising cell‐based strategy for treating cartilage‐related conditions such as OA, intervertebral disc degeneration and focal cartilage defects, especially given the limited self‐repair capacity of avascular cartilage tissue. Current research increasingly focuses on combining 3D culture techniques with bioactive scaffolds to enhance the efficiency of chondrogenic differentiation in both fundamental and translational studies [61, 62, 63].

### 2.3 Anti‐Inflammatory and Immunomodulatory Capabilities of BMSCs

Inflammation is a fundamental defensive response to chemical, mechanical, or microbial insults, aimed at eliminating threats and restoring tissue function [64]. BMSCs demonstrate significant therapeutic potential in inflammation‐related diseases through their potent anti‐inflammatory and immunomodulatory capacities, which are mediated via direct cell–cell contact and extensive paracrine signalling [65, 66].

BMSCs can modulate various immune cells including T cells, B cells, natural killer (NK) cells, macrophages, monocytes, dendritic cells and neutrophils [67, 68, 69]. Specifically, they suppress effector T cell (e.g., Th1 and Th17) proliferation and pro‐inflammatory cytokine secretion such as interferon‐γ (IFN‐γ) and IL‐17 while promoting regulatory T cells differentiation to enhance immune tolerance. They also reduce NK cell cytotoxicity and IFN‐γ production to prevent excessive immune attacks [70], and inhibit monocyte differentiation toward dendritic cells to attenuate inflammatory responses [71]. The immunomodulation occurs through multiple mechanisms including direct gap junction formation via connexin proteins (e.g., Cx‐43 and Cx‐45), which enables signal molecule transfer (e.g., cAMP) to suppress immune cell activation [72, 73].

The paracrine activity of BMSCs represents another major immunoregulatory mechanism, mediated through the secretion of cytokines and extracellular vesicles (e.g., exosomes transporting microRNAs, mitochondria and proteins) [74]. The key mediators include indoleamine 2,3‐dioxygenase (IDO), inducible nitric oxide synthase (iNOS), prostaglandin E2 (PGE2), interleukin‐6 (IL‐6), transforming growth factor‐β1 (TGF‐β1), TNF‐stimulated gene 6 protein (TSG6) and human leukocyte antigen‐G5 (HLA‐G5) [75]. For instance, inflammatory cytokines IFN‐γ induce IDO expression. IDO mediates immunomodulation through the inhibition of T cell function/proliferation, an increase in Treg numbers and a shift in the cytokine balance toward an anti‐inflammatory state (elevated IL‐10, suppressed IFN‐γ and TNF‐α) [76]. Similarly, key factors secreted by BMSCs—including IL‐6, hepatocyte growth factor (HGF) and interleukin‐1 receptor antagonist (IL‐1RA)—promote M2 macrophage polarisation while suppressing the pro‐inflammatory M1 phenotype and dendritic cell differentiation [77].

In bone‐related disorders, the immunomodulatory functions of BMSCs are particularly relevant. Osteoarthritis is associated with persistent low‐grade inflammation [78], fracture healing initiates with an inflammatory phase [79] and osteoporosis involves chronic inflammation within the bone microenvironment [80]. Through these coordinated mechanisms, BMSCs establish a sophisticated regulatory network that helps restore immune homeostasis in skeletal diseases, although the complexity of this network continues to present substantial research challenges.

### 2.4 Homing and Migration Capacity of BMSCs

The homing of MSCs refers to their targeted migration from the vascular system across endothelial barriers to specific injury sites, representing a crucial self‐repair mechanism in organisms [81]. The homing process of BMSCs involves multiple synergistic steps: the injured tissue releases ‘homing signals’, BMSCs undergo transendothelial migration and ultimately establish residence at the injury site. BMSCs express various functional chemokine receptors that facilitate this process, including CC chemokine receptors (CCR1, CCR7 and CCR9) and CXC chemokine receptors (CXCR4, CXCR5 and CXCR6). Their migration is further regulated by inflammatory cytokines (e.g., TNF‐α, IL‐1β, IL‐6 and IL‐8) and growth factors (e.g., VEGF‐A, FGF, PDGF‐AB, HGF, TGF‐β1, SDF and IGF‐1) [82, 83, 84]. The homing migration capacity of BMSCs facilitates targeted delivery to injured tissues and enhances therapeutic efficacy, forming the foundation for precision medicine.

In situ BMSCs play pivotal roles in skeletal development, remodelling and regeneration, underscoring their therapeutic significance for bone‐related disorders [85]. During early development, they function as direct progenitors for osteoblasts and chondrocytes, actively driving the formation and growth of bone and cartilage. Following the slowdown or cessation of skeletal growth, BMSCs can enter a state of reversible quiescence, serving as a long‐term reservoir that can be activated to proliferate and differentiate, thereby maintaining bone homeostasis through continuous remodelling [86]. Upon bone injury, these resident cells are mobilised to the site of damage, where they participate in the repair process by differentiating into osteoblasts and secreting regenerative factors [87, 88]. Crucially, culture‐expanded BMSCs retain these innate capabilities—particularly their osteogenic potential, niche‐supportive secretory activity and injury‐responsive homing ability. These key properties form the functional basis for their use as an attractive, physiology‐informed cell source for bone tissue engineering and regeneration strategies.

Researchers have explored the metabolic mechanisms of BMSCs in various scenarios, and an increasing number of key genes or factors have been identified. For example, a hierarchical response during the process of cartilage turning into bone in skeletal development has been discovered [89]; the key regulatory factor Ptip helps stromal cells remain in a quiescent state to maintain their vitality [90]; and STAT3 regulates skeletal development and guides osteogenesis to maintain bone homeostasis [91]; bone injury initiates a complex signalling cascade involving numerous cytokines and widespread gene expression changes, which spatiotemporally coordinate BMSCs proliferation and differentiation for repair [92]. Understanding these orderly core processes provides the essential theoretical basis and molecular targets for developing targeted therapies and novel regenerative approaches for bone disorders.

## 3 Applications of BMSCs in Bone‐Related Disorders

Leveraging their multifunctional properties, BMSCs have been extensively investigated for various bone‐related disorders, including osteoporosis, osteoarthritis, fracture repair and medication‐related osteonecrosis of the jaw.

### 3.1 Osteoporosis

Dysfunction of BMSCs plays a central role in osteoporosis pathogenesis, primarily manifested as cellular senescence, impaired osteogenic‐adipogenic balance and disrupted bone remodelling [93, 94, 95]. Restoring the osteogenic potential of BMSCs has thus emerged as a key therapeutic strategy. Current approaches include direct activation of osteogenic pathways (e.g., PI3K/AKT and Wnt/β‐catenin) via genetic editing or pharmacological intervention, often through overexpression of key regulators like *Runx2* [96, 97]. Concurrently, targeting cellular senescence by activating anti‐aging pathways (e.g., SIRT1 and AMPK) and enhancing autophagy improves BMSCs survival and differentiation capacity [98, 99]. Research indicates that the aging and functional decline of BMSCs are closely associated with autophagy. Activating the autophagy process can effectively promote cell survival, modulate energy allocation and reverse cellular aging, thereby creating favourable conditions for osteogenic differentiation [100, 101, 102, 103]. In osteoporosis, the chronic inflammatory microenvironment disrupts this bone balance [104]. BMSCs secrete anti‐inflammatory factors (e.g., IL‐10 and TGF‐β) that modulate the local immune microenvironment, suppressing osteoclast activation while simultaneously promoting their own osteogenic function to restore bone homeostasis [105, 106].

Notably, BMSC‐derived exosomes have demonstrated significant therapeutic potential. Exosomes secreted by BMSCs are rich in microRNAs (e.g., miR‐29a and miR‐146a) and cytokines, which coordinate bone formation through multiple mechanisms: modulating macrophage polarisation, enhancing angiogenesis and promoting osteogenic differentiation [107, 108, 109, 110]. Strategic pre‐treatment of exosomes, including physical stimulation, chemical induction and bioengineering approaches, can enhance exosomes' efficacy [111]. Moreover, engineering exosomes with bone‐targeting peptides enables precise regulation of bone metabolism, advancing targeted therapy for osteoporosis [112].

### 3.2 Osteoarthritis

OA is a degenerative joint disorder primarily managed through conservative approaches. Standard care combines physical therapy (e.g., muscle strengthening and electrotherapy) with pharmacological treatment (e.g., NSAIDs and corticosteroids) to relieve symptoms [113]. However, this approach only alleviates symptoms without halting disease progression. In end‐stage cases, surgical intervention such as joint replacement becomes necessary, which is nonetheless problematic due to the associated trauma and limited longevity of prostheses [114]. In contrast, BMSCs offer a regenerative strategy targeting the underlying disease mechanisms.

BMSCs exert multifaceted therapeutic effects in OA, including cartilage matrix repair, inflammation suppression and joint microenvironment modulation. And also they promote cartilage regeneration by modulating key pathways involved in pro‐inflammatory signalling, matrix degradation (via MMPs) and Wnt signalling [115, 116]. Chondrogenic differentiation of BMSCs—enhanced by microenvironmental cues (e.g., hypoxia and TGF‐β) or genetic modification (e.g., SOX9 overexpression)—stimulates the synthesis of type II collagen and proteoglycans, facilitating structural repair [117, 118]. The therapeutic effects are further mediated through extracellular vesicles (EVs) derived from preconditioned BMSCs, which contain various bioactive molecules that modulate the inflammatory microenvironment and activate chondrocyte autophagy to remove damaged organelles, thereby slowing cartilage degeneration [119, 120, 121]. While direct intra‐articular injection of BMSCs or EVs is commonly used, rapid clearance limits its efficacy [122]. Tissue engineering approaches address this by encapsulating cells or vesicles in biomimetic scaffolds (e.g., hyaluronic acid and composite hydrogels). These materials reduce immune clearance, mimic native cartilage mechanics and provide sustained release, supporting long‐term tissue restoration [123, 124, 125]. Advanced multi‐layered scaffold materials with distinct properties enable spatial functional partitioning and temporal regulation, achieving the dual effect of promoting neocartilage cartilage formation while simultaneously facilitating subchondral bone remodelling [126]. Collectively, BMSC‐based strategies represent a pivotal shift in OA treatment paradigms, transitioning from palliative relief toward functional restoration.

### 3.3 Fracture Healing

Fractures, caused by trauma or underlying bone fragility, are typically managed through surgical fixation. The healing process involves three sequential phases: inflammatory response, callus formation and bony callus remodelling, in which immune regulation and adequate vascular supply play critical roles [127]. Complications such as delayed union or non‐union may arise due to infection or impaired blood flow [128]. BMSCs contribute to fracture repair through multiple mechanisms. Upon transplantation, they promote polarisation of M2 macrophages and secretion of anti‐inflammatory factors (e.g., IL‐10 and TGF‐β), thereby attenuating excessive inflammation and establishing an osteogenesis‐favourable microenvironment [129]. Furthermore, through paracrine signalling in combination with growth factors such as PDGF and vascular endothelial growth factor (VEGF), BMSCs enhance angiogenesis via paracrine signalling, improving local nutrient supply and supporting bone regeneration [130]. Tissue engineering strategies further extend the therapeutic potential of BMSCs. Biomimetic scaffolds—for instance, hydroxyapatite‐collagen composites—loaded with BMSCs or derived vesicles provide mechanical support and osteoconductive signals while mimicking native bone structure [131, 132]. These scaffolds can also be functionalised with anti‐inflammatory drugs, bioactive ions (e.g., strontium), or signalling molecules to modulate immune responses and promote mineralisation [133, 134, 135]. Additionally, they facilitate the recruitment and osteogenic differentiation of endogenous BMSCs, thereby accelerating the healing process. While surgical fixation remains the current standard of care, BMSC‐based therapies represent a promising direction for enhancing biological repair and functional recovery.

### 3.4 Medication‐Related Osteonecrosis of the Jaw (MRONJ)

MRONJ is defined as the presence of exposed necrotic bone in the oral cavity, presenting with persistent and refractory symptoms lasting for more than 8 weeks, in patients who have not undergone radiotherapy. The disease involves numerous risk factors, including compromised immunity, trauma and viral infections, while it is most strongly associated with the use of anti‐resorptive agents, particularly nitrogen‐containing bisphosphonates (BPs). Current management predominantly relies on conservative non‐surgical approaches to slow disease progression, though these do not offer a definitive cure [136]. At present, BMSC transplantation combined with comprehensive therapy appears to be a promising therapeutic strategy for MRONJ [137, 138]. Research indicates that BMSCs exert significant therapeutic effects on MRONJ through synergistic multi‐mechanistic actions. In animal disease models, BMSC transplantation has been shown to treat MRONJ via immunomodulatory functions [138]. The therapeutic efficacy of BMSCs may be associated with intercellular mitochondrial transfer, which provides energetic support to damaged tissues and promotes cell survival [139]. Additionally, exosomes derived from BMSCs serve as crucial paracrine mediators, carrying various bioactive molecules that coordinately regulate bone homeostasis [140]. In an allogeneic transplantation model, BMSCs significantly reduced the incidence of osteonecrosis‐like lesions at tooth extraction sites in zoledronic acid‐treated rats [141], providing robust preclinical evidence. Although preclinical results are promising, the widespread clinical translation of BMSC‐based therapies still faces challenges and requires further high‐quality basic and clinical research to advance toward mature clinical implementation.

## 4 BMSC‐Based Therapeutic Strategies

**FIGURE 2:**
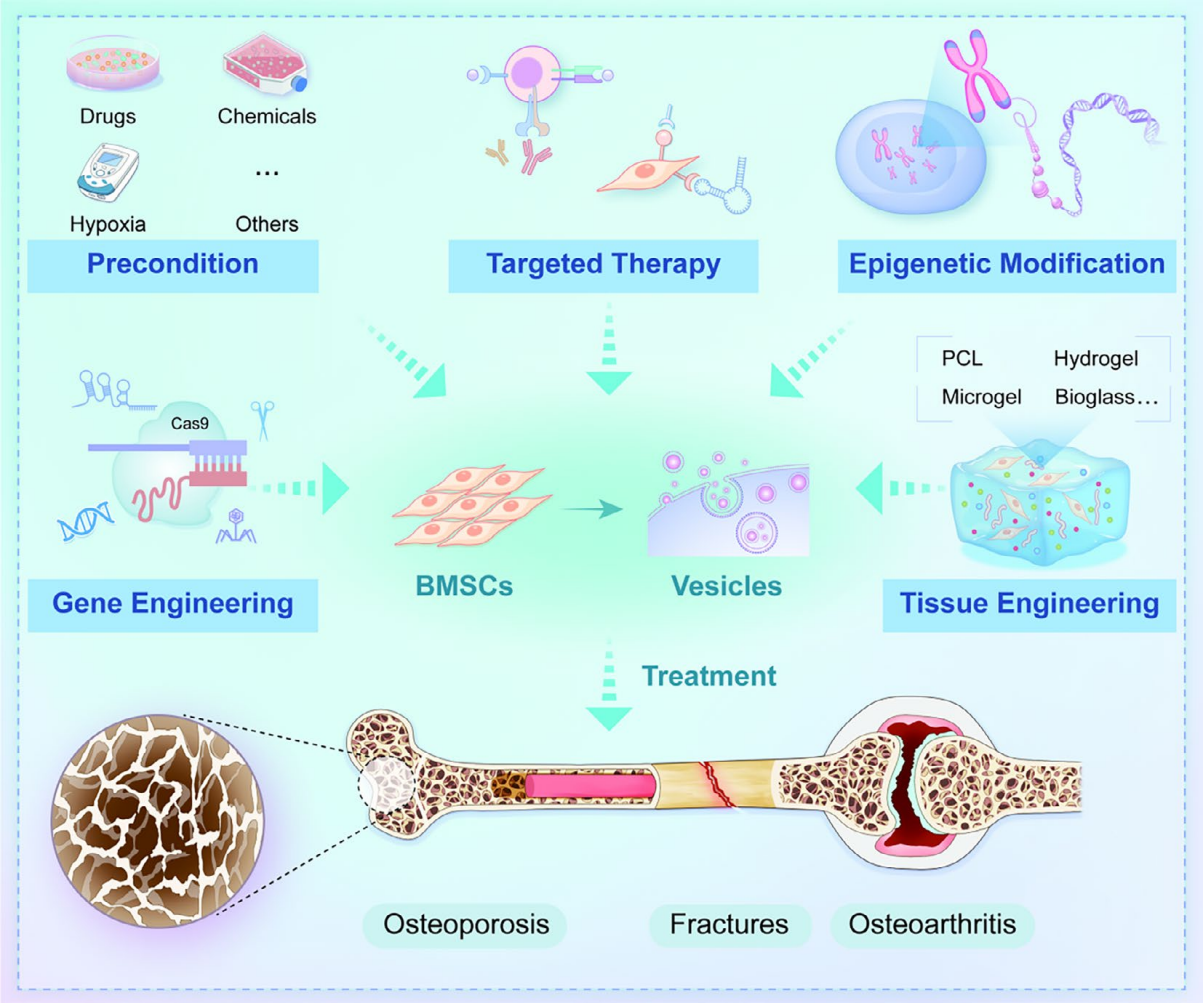
Engineered BMSCs and vesicles enhance therapy effects for bone diseases via multi‐strategic approaches.

To enhance the therapeutic efficacy of BMSCs and their derivatives, a range of modification strategies have been developed. These include genetic engineering, preconditioning, tissue engineering, vesicle engineering, targeted modification and epigenetic regulation. By optimising key cellular properties—such as proliferation capacity, differentiation potential, immunomodulatory function and homing ability—these approaches aim to overcome existing limitations in BMSC‐based therapies while maximising their inherent advantages for treating bone‐related diseases. The following sections provide a detailed examination of these strategies (Figure 2).

### 4.1 Genetic Engineering Modification of BMSCs

The clinical translation of unmodified BMSCs has been hampered by their relatively low therapeutic efficacy, significant side effects and unpredictable outcomes. To address these challenges, gene editing technologies (e.g., CRISPR‐Cas9, TALENs and zinc finger nucleases) and viral vectors (e.g., lentivirus and adeno‐associated virus) enable precise modification of specific genes in BMSCs, enhancing their osteogenic differentiation, proliferative capacity and homing ability, thereby presenting novel therapeutic avenues for treating bone‐related diseases [142].

The CRISPR‐Cas9 system, recognised for its high precision, efficiency and specificity, has been extensively utilised in functional genomics, gene therapy and the generation of gene‐edited animal models [143, 144]. In BMSCs, this technology facilitates multiplexed gene targeting to regulate stemness, senescence, migration and differentiation, while modulating cytokine secretion and improving therapeutic performance [145, 146]. For example, knocking out genes that inhibit adipogenic differentiation (e.g., *Pparγ*) or overexpressing of pro‐osteogenic genes (e.g., *BMPs* and *Runx2*) can significantly enhance the osteogenic potential of BMSCs [147, 148]. This approach has also enabled the development of immortalised BMSCs lines—for example, via Rosa26‐directed integration of SV40T—that maintain trilineage differentiation potential while circumventing senescence during long‐term culture [149].

Lentiviral and AAV vectors represent mature platforms for stable genetic modification of BMSCs. Lentiviral systems support lasting transgene expression, for example, Hmga1 overexpression via lentivirus activates the Wnt signalling pathway and promotes osteogenesis, highlighting its utility in osteoporosis intervention [150]. AAV vectors offer advantages in safety and immunogenicity, enabling direct in vivo delivery through intra‐articular or intraosseous injection for localised gene expression [151]. Nevertheless, the selection of an appropriate vector requires careful evaluation of transfection efficiency and expression persistence, which are pivotal for successful clinical translation [152].

### 4.2 Conditional Preconditioning

Conditional preconditioning—using chemical, pharmacological, or environmental stimuli—effectively modulates BMSC behaviour to enhance anti‐inflammatory, regenerative and adaptive functions, establishing it as a key strategy for treating osteoporosis and osteoarthritis.

#### 4.2.1 3D Culture Environment

3D culture environments, such as decellularised matrices, 3D‐printed biomaterials provide spatial architecture and cell–cell contact points that more effectively regulate BMSC phenotype, proliferation, migration and protein expression compared to traditional two‐dimensional cultures [153, 154, 155]. Yu et al. [156] demonstrated that 3D culture conditions significantly increase the yield and collection efficiency of EVs. These 3D‐derived exosomes effectively promoted osteogenic gene and protein expression (e.g., RUNX2, OCN, OPN, COL1A1, and ALP) in human BMSCs while enhancing cell proliferation, migration and anti‐apoptotic effects. As the cornerstone of bone tissue engineering, 3D culture systems facilitate BMSC‐based construction of biomimetic bone and cartilage tissues, providing critical structural and biological support for defect repair.

#### 4.2.2 Hypoxic Preconditioning

Hypoxic preconditioning augments BMSC angiogenic capacity, glucose metabolism, proliferation and survival by simulating physiological low‐oxygen niches [157]. The upregulation of hypoxia‐inducible factor‐1α (HIF‐1α) enhances autophagy and reduces apoptosis under osteoporotic conditions [158]. Liu et al. [159] demonstrated that hypoxic‐preconditioned BMSCs exhibit improved post‐transplant survival and neovascularisation, thereby optimising the local oxygen microenvironment during bone repair. Hypoxia also modifies EV cargo: Yang et al. [160] found that hypoxic BMSC‐EVs contain elevated levels of soluble factors and miRNAs that mitigate joint inflammation and enhance cartilage regeneration. Similarly, Xiao et al. [161] showed that apoptotic bodies from hypoxic BMSCs carry miR‐223‐3p, which suppresses osteoclast differentiation and alleviates alveolar bone loss. While promising, hypoxic protocols require standardisation and mechanistic validation for clinical translation.

#### 4.2.3 Pharmacological Preconditioning

Pharmacological agents enhance BMSC function through cytoprotection, anti‐inflammatory and pro‐differentiation effects. Melatonin mitigates oxidative stress and senescence, upregulates *Runx2* expression and supports mitochondrial function to promote bone regeneration [162, 163, 164, 165]. N‐acetyl‐l‐cysteine (NAC) pretreatment elevates glutathione levels, improving oxidative stress resistance and osteogenesis [166]. Statins (e.g., simvastatin) stimulate *Bmp‐2* expression and inhibit *Pparγ*, favouring osteogenic over adipogenic differentiation [167, 168, 169]; also, simvastatin acts as an ERα coactivator, suggesting utility in postmenopausal osteoporosis [170]. Natural compounds—including quercetin, naringin, icariin, tanshinone IIA and astragaloside IV—also promote osteogenesis and are widely investigated for bone metabolic disorders [171, 172, 173]. Together, these agents direct BMSCs toward osteoblastic commitment, offering a pharmacological means to counteract bone loss.

#### 4.2.4 Cell Co‐Culture Systems

Cell co‐culture systems enhance BMSC therapeutic efficacy by simulating physiological microenvironments through intercellular signalling, soluble factor exchange and organelle transfer. These interactions significantly improve BMSC functionality in bone regeneration.

Co‐culture of adipose‐derived stromal cells (ASCs) with BMSCs significantly upregulates pro‐angiogenic factors (e.g., VEGF and CXCL1), osteogenic factors (e.g., Wnt5a) and TGF‐β signalling pathway components in BMSCs, enhancing osteogenesis and angiogenesis [174]. Mitochondrial transfer represents a crucial mode of material exchange in co‐culture systems, influencing BMSC proliferation, migration and differentiation through modulation of energy metabolism, oxidative stress and cell fate determination. Mitochondria can be transferred via tunnelling nanotubes (TNTs), exosomes, or cell fusion mechanisms [11, 13, 175, 176, 177]. This process influences BMSCs through metabolic reprogramming, oxidative stress modulation and energy homeostasis regulation. In bone disorders, mitochondrial transfer maintains chondrocyte metabolism and promotes cartilage repair [178]. BMSCs engage in bidirectional mitochondrial exchange—donating mitochondria to protect damaged cells [179], and receiving macrophage‐derived mitochondria, which modulate BMSC osteogenic differentiation through alterations in glucose metabolism and oxidative stress responses [180].

Despite promising preclinical results, clinical translation faces substantial challenges. Technical limitations include inefficient mitochondrial isolation/delivery and stability issues, while safety concerns encompass potential tumourigenicity and immunological compatibility. Further understanding of dynamic exchange mechanisms, long‐term phenotypic effects and optimal culture parameters is needed. Future research should prioritise protocol standardisation, mechanistic elucidation and development of clinically viable delivery systems.

### 4.3 Tissue Engineering and Organoid Technology

Tissue engineering represents an innovative therapeutic strategy for bone repair and cartilage regeneration by integrating biomaterials, stromal cells such as BMSCs, and bioactive factors to construct functional biomimetic substitutes. The core concept lies in utilising biomaterials to simulate the in vivo microenvironment while synergising with the proliferative and differentiation capacities of BMSCs. Through controlled delivery of drugs, factors, or vesicles and by modulating material properties, the ‘scaffold‐cell‐factor’ system enables multidimensional regenerative therapy [181, 182, 183, 184]. For instance, nano‐multifunctional mineralised silk fibroin biomaterials demonstrate remarkable capabilities, including ROS scavenging, immunomodulation, pro‐angiogenesis and promotion of BMSC osteogenesis, which significantly enhance bone defect regeneration [185, 186, 187]. The field has advanced significantly with the development of biomaterials exhibiting high biocompatibility, tunable mechanical properties and processability, such as polycaprolactone (PCL), hydrogels, hydroxyapatite and bioglass [188, 189]. These materials provide structural and biochemical support for BMSCs, enabling sustained release of bioactive molecules to reshape the cellular microenvironment, suppress inflammation and direct cell fate (Table 1).

**TABLE 1:** Innovative materials and application advantages in bone tissue engineering.

Material types	Archetypal representative	Main characteristics	Application	References
Natural polymeric materials	Silk protein, chitosan, hydrogel, collagen, etc.	Biocompatibility, injectability, ease of modification, simulation of the extracellular microenvironment.	Cartilage regeneration and wound repair, suitable for cell adhesion and differentiation	[190, 191, 192]
Synthetic polymer materials	PCL, Polylactic acid (PLA), etc.	Highly malleable with controllable mechanical properties, enabling the construction of intricate 3D structures via 3D printing.	Scaffold framework for bone defect repair, providing mechanical support	[193, 194]
Inorganic biomaterials	Hydroxyapatite (HA), bioglass, etc.	Composition approximates bone minerals, promoting osteoblast adhesion, proliferation and differentiation and exhibiting osteoconductive properties.	Osteoporosis and bone defect filling, enhancing integration between implant materials and host bone	[195, 196]
Nano‐physical materials	Rare metal nanoparticles, carbon nanotubes (CNTs), graphene, etc.	Excellent antibacterial properties, mechanical properties and electrical conductivity, regulating the balance between osteogenesis and osteoclastism.	Bone defect repair, stress dispersion, promoting bone formation	[197, 198, 199]
Composite/multifunctional materials	Silk protein‐hydroxyapatite composites, DNA hydrogels, etc.	Integrating the advantages of multiple materials, it can simultaneously possess anti‐inflammatory, antibacterial and pro‐angiogenic functions.	Diabetic bone defects, infectious non‐union of bones, etc.	[200, 201]

Remarkably, organoids technology presents new opportunities for tissue repair. Organoids are defined as 3D structures formed through the self‐organisation of stromal cells. They comprise organ‐specific cell types and mimic key in vivo tissue features via processes such as cell sorting and spatially restricted lineage commitment [202]. In principle, this technology leverages the innate self‐organising capacity of stromal cells to generate highly biomimetic 3D micro‐tissues, providing an ideal biological foundation for bone and cartilage repair. However, significant challenges remain in constructing fully functional bone organoids. Major limitations include replicating the specific morphology and size of hard tissues, establishing functional vascular networks and accurately simulating the complex cellular composition, hierarchical architecture and biomechanical properties of native bone and cartilage [203].

Therefore, current studies increasingly favour an engineering strategy. This approach utilises biomaterials with excellent viscoelasticity and moldability—such as demineralised extracellular matrices (ECMs), silk fibroin, various hydrogels and hyaluronic acid—as scaffolds. These materials are used to pre‐construct a microenvironment that supports cell growth and differentiation, guiding the formation of osteochondral composite tissues that more closely resemble the physiological state and are more conducive to in vivo repair [204, 205, 206]. For example, Zhu et al. [201] developed dynamic DNA/gelatin methacryloyl (GelMA) hydrogels (CGDE) mimicking natural bone tissue hierarchy with appropriate mechanical strength and enhanced viscoelasticity. These hydrogels facilitate directional cell migration, vascular ingrowth and supporting spatiotemporal structures for dynamic mineralisation and tissue remodelling, thereby more readily facilitating the generation of bone organoid‐like microtissues. Tissue‐engineered materials capable of inducing angiogenesis constitute a novel strategy for constructing large‐scale bone organoids with intrinsic nutrient delivery functions, thereby providing a promising therapeutic approach for ischemic bone diseases [207]. The integration of BMSCs with advanced scaffold materials, coupled with the convergence of bone tissue engineering and organoid, establishes a synergistic platform with significant therapeutic potential. This approach offers a promising strategy for bone repair and regeneration in the treatment of bone defects, osteoporosis, fractures and other skeletal disorders [208].

### 4.4 BMSC‐Derived Vesicles

#### 4.4.1 Native Vesicles

BMSC‐derived vesicles (exosomes, microvesicles and apoptotic bodies) offer distinct advantages over cellular therapies by avoiding risks of vascular occlusion, tumourigenicity and immunogenicity. These vesicles represent a safer and more versatile cell‐free therapeutic alternative, circumventing limitations associated with stem cell therapy including embolism formation, aberrant differentiation, infection, infusion toxicity, immunogenicity, tumourigenicity and ethical concerns [209, 210]. The therapeutic potential of BMSC‐derived vesicles stems from their inheritance of cell membrane and cytoplasmic components from parent cells, endowing them with similar therapeutic effects in anti‐inflammation, immunomodulation and tissue regeneration. Functioning as intercellular messengers, these vesicles can transfer mRNA, miRNA, proteins and lipids to recipient cells, influencing diverse biological processes including cell metabolism, survival, proliferation and immune responses [211, 212, 213, 214, 215]. In osteoarthritis models, BMSC‐derived exosomes were shown to counteract IL‐1β‐mediated inhibition of chondrocyte proliferation and migration, promote cartilage repair and extracellular matrix synthesis [121]. Apoptotic bodies have been found to maintain bone homeostasis by activating the Wnt/β‐catenin pathway in BMSCs to promote osteogenesis, offering novel insights for osteoporosis treatment [216]. The nanoscale characteristics of vesicles enable them to breach biological barriers, penetrate the minute pores of bone hard tissue and the dense matrix of cartilage tissue more efficiently and directly reach the site of injury to exert their effects.

#### 4.4.2 Engineered Vesicles

Despite their remarkable therapeutic potential, native vesicles face challenges including instability, low yield and suboptimal isolation efficiency. Optimisation of culture conditions and preconditioning strategies can significantly enhance MSC‐derived exosomes production and functionality [217, 218]. For example, 3D culture systems that better mimic the in vivo microenvironment have been shown to dramatically increase exosomes yield from BMSCs [219, 220, 221]. Similarly, hypoxic conditions boost exosomes release and modify their cargo composition (e.g., miRNA, RNA, proteins, lipids, growth factors and cytokines), thereby enhancing pro‐angiogenic, pro‐proliferative, pro‐migratory, anti‐inflammatory, antioxidant and anti‐apoptotic capacities, ultimately influencing diverse physiological processes including cell development, immunity, tissue homeostasis and diseases [222, 223].

More significantly, on the one hand, isolated exosomes serve as cargo carriers capable of passively or actively loading drug molecules: lipophilic drugs coexist with exosomes and are passively absorbed by them via concentration gradients, while hydrophilic drugs can be incorporated into exosomes through methods such as electroporation, ultrasonication, freeze–thaw cycles, extrusion, saponification and freeze–thaw cycles [224, 225, 226]. Exosomes significantly enhance drug utilisation compared to other administration methods [227]. On the other hand, the selective targeting of exosomes can be improved through surface modification, either by artificially adding receptors to vesicle membranes or using covalent modifiers to conjugate targeting molecules to the outer membrane, thereby enhancing tissue‐specific accumulation [228]. Labelled vesicles can also be observed in vivo via various detection techniques to monitor metabolic processes, distribution and tissue targeting, thereby facilitating the investigation of the response to vesicles and physiological metabolic processes [229, 230].

Synergistic approaches are also emerging. The fusion or co‐administration of vesicles derived from different cell types has demonstrated synergistic benefits. For example, co‐transplantation of BMSCs and endothelial cell‐derived membrane vesicles has been shown to mutually enhance osteogenesis and angiogenesis [231, 232]. Engineered vesicles have demonstrated superior performance in various disease models. For instance, dexamethasone‐loaded BMSC exosomes with surface modifications exhibited enhanced anti‐inflammatory effects while minimising systemic side effects in osteoarthritis treatment [233].

Despite promising results, clinical translation requires standardised production protocols, comprehensive safety evaluation and scalable manufacturing systems. Future efforts should focus on overcoming these translational challenges while expanding the therapeutic potential of engineered vesicle platforms.

### 4.5 Targeted Therapeutic Strategies

Targeted therapy employs precise interventions directed at specific molecular anchors, thereby enhancing therapeutic efficacy and specificity. The targeted delivery of BMSCs and their derived vesicles in vivo can significantly improve utilisation efficiency and treatment outcomes. Commonly used targeting molecules include aptamers, peptides and small molecular probes, with modification mechanisms based on specific recognition and biocompatible conjugation.

Aptamers, consisting of short nucleotide or peptide sequences, exhibit remarkable specificity in recognising targets ranging from small molecules to entire cells. Various molecular probes such as carbohydrates, short peptides, extracellular domains of cell membrane receptors and oligonucleotides can serve as targeting moieties for cell surface modification with high specificity and affinity [234, 235]. Furthermore, peptides enable precise control of amino acid composition and sequence. Through hydrophobic interactions between cell bilayers and peptide‐anchoring modules or covalent conjugation, engineered exogenous modifications of stromal cell surfaces can be achieved, providing appropriate signal transduction to enhance MSC homing capacity [236].

Numerous studies have demonstrated the successful application of aptamer‐conjugated tissue engineering for bone and cartilage regeneration. The specific recognition and binding capabilities of aptamers effectively recruit BMSCs to defect sites, where they can be loaded with bioactive factors to promote either chondrogenic differentiation for cartilage repair or osteogenesis for bone regeneration [237, 238]. Notably, targeted intervention at senescence sites can counteract stromal cell aging to promote bone regeneration. Wang et al. [239] identified specific cell surface markers of senescent MSCs and then developed a novel nanoplatform for dual delivery of resveratrol (RSV, a SIRT1 promoter) and nicotinamide riboside (NR, an NAD^+^ precursor). This senescence‐targeted, NAD^+^‐dependent SIRT1 activation system effectively reversed cellular senescence and promoted osteogenesis, demonstrating significant potential for repairing age‐related bone defects. BMSCs and their secreted vesicles serve as ideal nanocarriers for drug delivery. Inorganic nanoparticles (NPs) can be attached to the surface of cells or vesicles via electrostatic/hydrophobic interactions, covalent bonding, or physical adsorption. This conjugation strategy effectively anchors therapeutic cargo to these biological carriers [240]. This targeted delivery approach combines the biological advantages of stromal cells with the precision of nanotechnology, creating a powerful platform for regenerative medicine applications [241].

### 4.6 Epigenetic Modifications of BMSCs

Epigenetics refers to reversible and heritable changes in gene expression that occur without alterations to the nuclear DNA sequence. These modifications include DNA methylation, RNA methylation, histone modifications, microRNAs (miRNAs) and non‐coding RNAs, which play critical regulatory roles in regulating BMSC differentiation, proliferation and senescence [242, 243]. Epigenetic modifications are closely linked to the development and progression of orthopaedic diseases [244, 245, 246], and ongoing research into these processes has deepened our understanding of BMSC metabolic activities in disease contexts.

#### 4.6.1 DNA and RNA Methylation in BMSCs

DNA methylation is a dynamic process regulated by the coordinated actions of DNA methyltransferases (DNMTs), demethylases (TETs) and modifications such as 5‐methylcytosine (5mC) and 5‐hydroxymethylcytosine (5hmC). This process critically influences BMSC lineage commitment by modulating the expression of key transcription factors [247, 248]. For instance, DNA methylation influences BMSC differentiation—both osteogenic genes (e.g., *Runx2* and *Osterix*) and adipogenic genes (e.g., *Pparγ* and *Cebp*) [249]. Dysregulation of this system contributes to disease, aberrant DNMT expression in osteoporosis pathogenesis can suppress GPX4 and promote ferroptosis in osteoblasts [250].

RNA methylation, particularly m^6^A modification exerts complex regulatory effects on mRNA, mediating its translation efficiency, stability and degradation. As the most abundant mRNA modification, m^6^A methylation is dynamically and reversibly regulated through the coordinated spatiotemporal actions of ‘writer’ enzymes (e.g., METTL3/METTL14), ‘eraser’ enzymes (e.g., FTO, ALKBH5) and ‘reader’ proteins (e.g., YTHDF family), ultimately influencing cellular metabolic activities [251, 252, 253]. Numerous studies have demonstrated that RNA methylation‐related genes play crucial roles in regulating osteogenesis. For instance, knockdown of *Mettl3* or *Ythdf3* impairs bone formation [254, 255, 256, 257], while its overexpression can rescue bone loss by activating pro‐osteogenic pathways like Wnt signalling pathway [38]. The ‘eraser’ FTO modulates autophagy and energy metabolism to alleviate meniscal degeneration and osteoarthritis, it may represent a target for preventing cartilage damage [258].

#### 4.6.2 Histone Modifications

Histone proteins undergo a vast array of post‐translational modifications (e.g., methylation, acetylation, phosphorylation, lactylation, citrullination, crotonylation, succinylation, SUMOylation, propionylation, butyrylation, 2‐hydroxyisobutyrylation and 2‐hydroxybutyrylation) that alter chromatin structure and gene accessibility [259]. These modifications have been shown to play significant roles in various disease processes, though many mechanistic details remain to be elucidated.

Researches on histone modifications in BMSCs have gained significant momentum, revealing their critical involvement in regulating essential cellular processes such as differentiation, migration and proliferation [260, 261]. For instance, SIRT3, an NAD^+^‐dependent histone deacetylase, counteracts age‐related bone loss. The S‐sulfhydration of SIRT3 stabilises heterochromatin structure and maintains mitochondrial homeostasis, thereby effectively counteracting BMSC senescence [25]. Metabolite‐driven modifications provide a direct link between cellular metabolism and epigenetics. Endothelial cell glycolysis promotes BMSC osteogenesis through histone lactylation modifications. This process, coupled with elevated serum lactate levels, has been shown to ameliorate osteoporotic conditions [262].

#### 4.6.3 Non‐Coding RNAs (ncRNAs)

Non‐coding RNAs (ncRNAs) regulate gene expression through multiple mechanisms, including transcriptional and post‐transcriptional regulation. Among the most extensively studied ncRNAs are long non‐coding RNAs (lncRNAs), circular RNAs (circRNAs) and microRNAs (miRNAs), which significantly influence cellular differentiation, proliferation, senescence and other metabolic activities [263, 264, 265]. Their regulatory roles are complex, they can simultaneously target multiple genes or pathways, or the same ncRNA may exert opposing effects [266]. For example, miR‐21 promotes tissue repair in inflammatory conditions but facilitates metastasis in tumours [267, 268]. LncRNAs can mediate crosstalk between angiogenesis and osteogenesis by sponging miRNAs or enhancing VEGF expression, thereby promoting osteogenic differentiation [269]. However, certain lncRNAs have also been shown to inhibit osteogenic differentiation [270].

Recent years have witnessed the discovery and investigation of emerging ncRNA members, including enhancer RNAs (eRNAs), tRNA‐derived small RNAs (tsRNAs) and piwi‐interacting RNAs (piRNAs), expanding our understanding of ncRNA regulatory networks [271, 272]. However, research on these ncRNAs in BMSCs remains limited, indicating an important area for future exploration.

In summary, epigenetic modifications in BMSC metabolism form an intricate and interconnected network. For instance, METTL3 can influence the stability of lncRNAs, thereby affecting stromal cell osteogenic differentiation. Simultaneously, m^6^A methylation modifications can alter miRNA maturation processes, subsequently impacting BMSC osteogenesis [273]. The discovery of ncRNA transport via extracellular vesicles not only expands the functional repertoire of vesicles but also reveals additional mechanisms of ncRNA metabolic regulation [274, 275]. These findings highlight the complex, multi‐layered regulatory systems governing BMSC behaviour and present new opportunities for therapeutic intervention in bone‐related disorders.

## 5 Application Limitations of BMSCs in Bone‐Related Diseases

Despite their considerable potential in regenerative medicine, the clinical application of BMSCs is still constrained by several limitations [276]. These challenges can be categorised into inherent cellular constraints, microenvironmental barriers post‐transplantation and hurdles in clinical translation.

A primary limitation is the cellular heterogeneity of BMSCs. Populations designated as BMSCs comprise multiple subpopulations with distinct phenotypes and functional characteristics [277]. This heterogeneity, influenced by donor age, cell source and culture conditions, directly impacts expansion potential and stability [278, 279]. Furthermore, BMSCs face challenges in maintaining their stemness. With prolonged culture or increased donor age, they may lose pluripotency, exhibit hyperproliferation, or undergo undesired differentiation into non‐target cell types, compromising therapeutic efficacy and safety [280, 281]. Additionally, genetic instability poses a significant safety risk. Long‐term culture can lead to chromosomal abnormalities, increasing the potential for tumourigenesis or functional abnormalities [282, 283, 284]. These intrinsic cellular constraints fundamentally limit the reliability and scalability of BMSC‐based therapies.

Upon delivery, transplanted BMSCs encounter a hostile injured niche that severely compromises their survival and function. The hostile microenvironment at the injury site—characterised by hypoxia, inflammation and immune responses—severely restricts cell survival [285]. Moreover, the homing capacity of BMSCs is often insufficient. Intravenous administration suffers from a first‐pass effect, and the decreased expression of homing‐related receptors (e.g., CXCR4) impairs targeted migration. Furthermore, inflammatory factors and local drug concentrations within the damaged niche further disrupt homing efficiency [84, 167].

The path to clinical application is fraught with practical challenges. The irregular nature of bone defects demands personalised biomaterial carriers, while monitoring cell distribution post‐implantation remains technically difficult [286]. The high costs and standardisation issues present major bottlenecks. BMSC preparation, expansion and genetic modification are expensive processes. Obtaining autologous cells via bone marrow aspiration has low patient acceptance, while allogeneic therapies require rigorous donor screening, for which standardised production and quality control systems are still underdeveloped [287]. Finally, predicting therapeutic outcomes is complex, as efficacy is influenced by disease types, patient‐specific factors and the microenvironment, making clinical trials difficult to design and interpret. Although preliminary human trials are promising, comprehensive safety validation is required before widespread clinical use [288].

## 6 Conclusion

Stem cell therapy, as a highly promising cellular treatment approach in contemporary medicine, has pioneered novel pathways for addressing bone tissue‐related disorders. Bone marrow mesenchymal stromal cells (BMSCs), serving as pivotal osteogenic precursor cells, play a decisive role in bone tissue repair and regeneration research. Extensive research has elucidated the cellular proliferation, differentiation and metabolic activities of BMSCs. Through in‐depth exploration of their gene expression and regulatory mechanisms, intercellular interaction patterns, vesicular secretory functions and signalling pathways, they have progressively elucidated the core regulatory principles governing BMSCs in the pathogenesis, clinical management and tissue regeneration processes of bone‐related disorders such as osteoporosis, osteoarthritis, fractures and medication‐related osteonecrosis of the jaw. These fundamental research findings have not only refined the theoretical framework for BMSC‐based therapies but also provided robust experimental evidence and scientific support for subsequent clinical translation of cell therapies.

However, the inherent complexity of biological systems presents significant challenges for the clinical application of BMSCs. On the one hand, the precise regulatory mechanisms governing the signalling networks during BMSC development remain incompletely understood, with abnormalities at any stage potentially impairing their functional efficacy. On the other hand, the differentiation outcomes and functional states of BMSCs in vitro exhibit marked discrepancies from their behaviour within the authentic tissue microenvironment in vivo, making it difficult to accurately predict their therapeutic effects in the body. Simultaneously, the maintenance of BMSC activity and functional plasticity is constrained by multiple factors including donor age, culture conditions and the pathological microenvironment. Collectively, these issues limit the stability and reliability of BMSCs' clinical outcomes.

To overcome these limitations, researchers have actively integrated multidisciplinary techniques, developing diversified intervention strategies that significantly enhance the therapeutic efficacy of BMSCs and their derivatives. For instance, to address core challenges such as low cell survival rates, poor homing efficiency and functional instability, various strategies have been developed to improve the capabilities in proliferation, differentiation, migration and survival, while also enhancing the functionality of their derivatives. This multi‐layered approach—spanning from cellular to non‐cellular therapies—collectively elevates the application of BMSCs in the treatment of bone‐related diseases. Genetic engineering techniques, utilising tools such as CRISPR‐Cas9, enable precise editing of key genes to optimise the homing and differentiation capabilities of BMSCs. Pre‐treatment technologies, employing methods like hypoxia induction and factor stimulation, enhance anti‐apoptotic and paracrine functions of BMSCs. Tissue engineering techniques, combined with 3D printing, create biomimetic scaffolds that provide mechanical support and a differentiated microenvironment for BMSCs. Targeted intervention techniques enable precise delivery of BMSCs and their derivatives; epigenetic regulation further unlocks therapeutic potential by modulating gene expression patterns. Even if BMSCs undergo apoptosis after transplantation, engineered cells can be transformed into engineered apoptotic vesicles within the body. As mentioned above, these vesicles inherit the superior functional properties of the cells, exert therapeutic effects and compensate for the functional loss of the apoptotic cells [289]. Thus, non‐cellular therapy can effectively serve as a functional continuation of cell therapy in vivo. While the translational dynamics from in vitro studies to in vivo environments inevitably present discrepancies, navigating and reconciling these differences represents a critical and indispensable reality for advancing the clinical translation of BMSC‐based therapies.

Given that a single strategy often has inherent limitations, the integration of multiple strategies generates synergistic effects through functional complementarity and has become a mainstream research paradigm. For example, combining tissue engineering materials with vesicles allows fibrin hydrogels to maintain and slowly release exosomes derived from BMSCs, thereby regulating chondrocyte function [290]; similarly, cobalt‐containing bioactive glass scaffolds can provide structural support while simulating a hypoxic microenvironment, promoting angiogenesis and osteogenesis [291]. These combined approaches significantly enhance the therapeutic effects of BMSCs and their vesicles. The multi‐strategy integration targeting BMSCs to compensate for shortcomings represents the optimal approach in current development. Naturally, the integration of multiple strategies also presents new challenges, including increased process complexity, stringent technical compatibility requirements and inconsistent safety assessment standards, which will demand higher technical development standards.

Moving forward, as these technologies undergo continuous innovation and deep integration, BMSCs will undoubtedly play an increasingly pivotal role in precision treatment for bone tissue‐related disorders, propelling cell therapy toward new heights.

## Author Contributions

X.L., D.T., Q.L. and J.X. conceptualised, supervised and revised the manuscript. X.L., D.T. and L.Y. conducted the literature review and drafted the paper. Q.T. and Q.Y. helped prepare the manuscript. D.T. and J.X. provided funding for the manuscript. All co‐authors have reviewed and approved this version of the manuscript.

## Funding

This work was supported by National Natural Science Foundation of China (82370938); Science and Technology Department of Sichuan Province (2024NSFSC2087); Open Project of State Key Laboratory of Oral Diseases (SKLOD2025OF05); Science and Technology Project of Health Commission of Sichuan Province (24WXXT11); Scientific and Technological Project of Luzhou (2025MYF002, 2025MYF024); The Youth Innovation Project of Sichuan Medical Association (Q20250047); 2023 Key Project of The Affiliated Stomatological Hospital of Southwest Medical University (2023Z02); The 2025 Doctoral Research Initiation Project of the Affiliated Stomatological Hospital of Southwest Medical University (2025BS04); Stomatological Special Fund of Southwest Medical University (2024KQZX08, 2024KQZX02).

## Conflicts of Interest

The authors declare no conflicts of interest.

## Data Availability

Data sharing not applicable to this article as no datasets were generated or analysed during the current study.
